# It Began in Ponds and Rivers: Charting the Beginnings of the Ecology of Fish Cognition

**DOI:** 10.3389/fvets.2022.823143

**Published:** 2022-02-03

**Authors:** Susan D. Healy, B. Wren Patton

**Affiliations:** ^1^Centre for Biological Diversity, School of Biology, University of St Andrews, St Andrews, United Kingdom; ^2^Department of Ecosystem Science and Management, Penn State University, State College, PA, United States

**Keywords:** cognitive ecology, fish, homing pigeon, navigation, predation, spatial cognition

## Abstract

But fish cognitive ecology did not begin in rivers and streams. Rather, one of the starting points for work on fish cognitive ecology was work done on the use of visual cues by homing pigeons. Prior to working with fish, Victoria Braithwaite helped to establish that homing pigeons rely not just on magnetic and olfactory cues but also on visual cues for successful return to their home loft. Simple, elegant experiments on homing established Victoria's ability to develop experimental manipulations to examine the role of visual cues in navigation by fish in familiar areas. This work formed the basis of a rich seam of work whereby a fish's ecology was used to propose hypotheses and predictions as to preferred cue use, and then cognitive abilities in a variety of fish species, from model systems (Atlantic salmon and sticklebacks) to the Panamanian *Brachyraphis episcopi*. Cognitive ecology in fish led to substantial work on fish pain and welfare, but was never left behind, with some of Victoria's last work addressed to determining the neural instantiation of cognitive variation.

## Introduction

In the past three decades, our understanding of what fish can perceive, attend to, learn, and remember has gone from little and assuming less, to the inclusion of fish in any course on animal cognition. Gone is the mention of the three-second memory of the goldfish, in is the awareness of pain, the ability to count, navigational abilities rivaling those of a homing pigeon, and much more.

Indeed, it was work with homing pigeons that first brought about some of the major changes in our current understanding of fish cognition. This is because much of the responsibility for our deepening understanding of the cognitive capacities of fish lies with Victoria Braithwaite, and her story starts with a flock of pigeons. Braithwaite's contributions come from asking questions about fish cognition in the context of their ecology and evolution, and how natural selection might have shaped their cognitive abilities. In this review, we therefore have two aims: first and foremost, to examine the impact of Victoria Braithwaite's work on current understanding of orientation and navigation in fish and other vertebrates, and second, to reflect on how bringing this adaptationist view of fish cognition brought fish into the mainstream of a field previously dominated by mammals and birds. Our particular focus on Braithwaite's work is unabashedly firstly as a memorial to our friend and colleague whose untimely death in 2019 we mourn but also because we contend that her work was pivotal in the establishment of fish as mainstream, even conventional, in work on animal cognition.

## Navigation and Visual Landmarks

That work began, as alluded to above, with the avian model for navigation: homing in pigeons. By the late 1980's it was firmly established that birds, among other animals, used all manner of cues to guide their journeys, long or short. Homing pigeons formed the basis for a large part of the “real-world” experimental investigation into vertebrate navigation. One reason for their popularity as a model was that multiple features could be readily manipulated including their rearing, housing, transport, sensory input, and experience. However, there had been relatively little investigation into their use of landmarks and other visual cues. This was perhaps because of the famous 1970's experiment in which pigeons returned to their home loft even when wearing frosted lenses (1), leading to the belief that visual cues were not important to homing pigeon navigation. And so it took until the early 1990's before Braithwaite and Guilford (2) showed that even 5 min of viewing a familiar landscape prior to release was sufficient to reduce the time it took homing pigeons to return to their loft in comparison to control birds confined in a box with opaque walls. A subsequent experiment confirmed that the recognition of familiar landmarks visible at the release site was the key to the difference in homing time and not other factors such as a reduction in confidence of the pigeons in homing. In this latter experiment, birds homed from familiar and unfamiliar release sites and were allowed to view or not view the landscape for 5 min prior to release. Only the birds allowed visual access to the familiar site prior to release homed faster (3).

These data, followed by confirmatory experiments in the next few years (3–5), showed that visual information (landmarks) could be important in enabling pigeons to home, in addition to the other cues (especially magnetic and olfactory) that had long been the focus of the pigeon homing community. More than two decades on, the degree to which visual landmarks influence the route a pigeon takes toward its home loft and the speed at which it does so continues to be debated and elucidated [e.g., (6–9)]. Some of the questions that arose from those early pigeon release data, such as the importance of landmarks at points later in a journey rather than just at the starting point, had to wait until the development of appropriate technology such as GPS tags for tracking animals [e.g., (10, 11)]. But the early data also initiated an interest in the role of other visual information that homing pigeons might use, such as the identity (12) and experience [e.g., (13)] of their flock partners. These studies also laid the foundation for the examination in other species of the role played by visual information in navigation in other species. Alongside birds and mammals, attention began to be paid to fish too.

It is a common observation in navigation texts that fish can perform remarkable navigational feats. The most famous examples come from data showing that migratory fish like salmon successfully return from the open ocean to their home stream by using olfactory cues (14–16). Even non-migratory fish are known to be expert navigators: ironically, given the popularity of the myth of their three-second memories, there are century-old data showing successful learning and navigation of a maze by goldfish (17). However, much like with homing pigeons, the body of research on memory and cue use in fish [e.g., (18–20)] contained surprisingly few attempts to investigate their use of *visual* cues. This omission is striking, considering that visual cues have always been the focal cue type for spatial cognition work in rats and mice. This is perhaps because visual cues are so much more readily manipulable by human researchers, dependent as we are on our visual capacities. Braithwaite's early fish experiments were among the first to ask what visual cues fish might use to navigate around a familiar area. In her first experimental manipulation on visual cue use by fish in 1996, using a flume tank and colored plastic Lego bricks as landmarks, Braithwaite and her co-authors showed that Atlantic salmon *Salmo salar* could use conspicuous visual cues to track a moving resource (21).

A second experiment contained within that 1996 paper showed that when conspicuous visual cues were no longer available, the fish would switch to another preferred cue type for navigation. This secondary cue was probably chemosensory. But it was clear that fish differed in their preferred cue type, as only some fish switched to the chemosensory option when the conspicuous visual cues were no longer available. This evidence that the salmon might use more than one cue type, or have a hierarchy of cues, also echoed work from homing pigeons. Furthermore, the variation among the fish in cue preference and in performance on the task prefigured the current enthusiasm for understanding differences among individuals [e.g., (22)] and coping strategies [e.g., (23)]. The simplicity of the experimental method and the salmon data themselves formed the basis for work that continues today. Some of that work involves identifying the kinds of information used by fish when moving within and between locations, familiar or novel, just as Braithwaite et al. (21) did 25 years ago. There is plenty of scope for such work, as shown by the large and growing number of species examined. An incomplete list of this species includes French grunts *Haemulon flavolineatum* (24), freshwater stingrays *Potamotrygon motoro* (25), Amarillo fish *Girardinichthys multiradiatus* (26), and rainbowfish *Melanotaenia* spp. (27). Perhaps not surprisingly (but one still has to collect the data), it is now typical to find that these fish, just like homing pigeons and salmon, have a hierarchy of cues when navigating [e.g., (24, 28, 29)].

Rather amazingly, however, the spatial movements of which fish are capable continue to surprise. For example, the three-spined stickleback *Gasterosteus aculeatus* is familiar to behavioral ecologists as a model for sexual selection [e.g., (30)] and speciation (31) among others, but it was not until 2013 that its ability to home after displacements of up to 180 m was demonstrated (32). Likewise, the ability of female cardinal fish *Apogon notatus* to return to the exact location of the territory they had held up to 6 months earlier could still be described as remarkable in 2010 (33). The apparent surprise in the demonstrations of the navigation abilities of these fish put us in mind the time it took to refute the belief that goldfish have memories older than 3 s: the idea that fish have any degree of capaciousness to their learning and memory abilities seems to have taken a long time to really take hold (34).

## Cognitive Ecology and Cue Use

Braithwaite's work on cue use in fish was, however, even more influential beyond the narrow focus of cue use and memory capacity in different fish species. Indeed, this work was one of the forerunners to the field we now refer to as ‘cognitive ecology’ (35). Around the middle 1980's and into the early 1990's behavioral ecologists began to ask questions about animal cognition that differed from the questions typically asked by experimental psychologists. Where the previous questions had included the nature of associative learning, whether timing is scalar, and the difference between working and reference memory, researchers in this new field asked whether and how cognitive abilities might have been shaped by natural selection. One of the first of these questions was whether spatial memory abilities are better when a species' ecology appears to depend heavily on spatial memory. One, now textbook, example centered around asking whether food-storing species had better spatial memory than did species that do not store food [e.g., (36, 37)]. Although a convincing demonstration of a difference between storers and nonstorers in spatial cognition took some years and multiple experiments [e.g., (38, 39)], both correlational and experimental data showed that (a) food storers had a larger hippocampus (the region of the vertebrate brain heavily involved in spatial information processing) than did nonstorers [e.g., (40, 41)], and (b) damage to the hippocampus in food storers reduced their ability to retrieve their stores and to solve spatial memory problems (42, 43).

These data set the scene for Braithwaite to bring together two worlds: cognitive ecology and fish. For the first set of experiments, Girvan and Braithwaite (44) chose to ask how the ecology experienced by three-spined sticklebacks *Gasterosteus aculeatus* was related to their performance on a spatial learning task. To do this, they used populations from a highly variable environment (a river) and contrasted them with populations from a stable environment (a pond). The task came in two versions, both using a linear maze in which the fish were trained to swim from the release compartment to the end of the maze for a food reward, through a series of choices (e.g., a set of open or closed doors). In one maze the route was marked by a visual landmark (a plant) at each of the correct decision points, while in the other maze there were no visual landmarks. The hypothesis was that fish from a stable environment might be more likely to use visual landmarks, whereas fish from a less visually stable environment might be more likely to rely on movement cues when orienting themselves in their habitats. Although the data did not entirely neatly dovetail with these predictions, the sticklebacks from two different ponds did take longer to learn how to navigate the maze when there were no landmarks than did the fish from the river populations. In addition, fish from one of the river populations took longer than fish from the other three populations to relearn the maze when the sequence of choices was reversed, which was consistent with these fish having learned a pattern of turns for successfully navigating the maze.

Much like the earlier food-storing work on birds [e.g., (45)], this experiment showed immediate support for a relationship between the ecological demands of the habitat and cue preferences, with the added flourish of the difference being within, rather than between, species. Although this work was soon cast in an adaptive framework (46), and indeed was consistent with that framework, it did not actually yet demonstrate differences in memory among the populations. Furthermore, one should always be aware that a wide variety of factors can and do affect the motivation of an animal to pay attention to, to learn or to remember an object, location, event or other. This difference in motivation or attention can result in an animal performing in such a way that looks poorer, or better than another. If an animal does not pay attention or does not value the reward, similarly to another individual, testing on a cognitive task is not occurring on a level playing field (47).

Since these early experiments, a multitude of experiments using ecology to predict cue use and cue preferences have ensued in birds, fish, and other taxa. In some, ecology does seem to explain those preferences, while from other experiments we have learned more about yet other ways in which context can affect test outcomes. For example, there are multiple experiments showing that when both color and spatial cues are available, nonstoring birds do not have a cue preference whereas storing birds prefer to use the spatial information. In a typical instance, a one-trial associative memory task, nonstoring blue tits *Cyanistes caeruleus* and jackdaws *Corvus monedulus* had no cue preference when the locations were specified by visual or spatial cues (48), while food-storing marsh tits *Parus palustris* and jays *Garrulus glandarius* preferred to visit the location specified by spatial cues. And yet, animals can learn to shift from using one cue to another even across the course of an experiment. For example, in another associative memory task, nonstoring great tits were trained to find food in the same location on 10 consecutive visits, always covered by a cloth flap of the same color. When given the choice between the familiar location covered with a cloth flap of a different color or a new location with a cloth flap of the familiar color, the nonstorers overwhelmingly chose the familiar location (i.e., using spatial cues) rather than the cloth flap of a familiar color (49). As seen in a variety of tests of spatial learning including rats in a Morris water maze, sticklebacks in a T-maze, and wild hummingbirds in the field, stability of cues seems to be important, and spatial cues are very often more stable than are visual cues (50–52).

In other cases, the relationship between ecological context and cue preferences is less obvious. One example is an experiment demonstrating the preference of two ecotypes of a facultative Caribbean cleaning goby *Elacatinus prochilos* for spatial over pattern cues (53). In that experiment, there were two ecologically-based predictions: (1) that cleaning gobies would perform better in a task relying on pattern cues, because the task (identifying a pattern on a plate) was analogous to deciding which clients to clean, while (2) sponge-dwelling gobies would perform better on a spatial task. In fact, both species did well on the spatial task and poorly on the pattern-cued task. The authors could only speculate as to the meaning of these results, but these data give notice that predictions about cue use may well test a researcher's understanding of the key attributes of the ecological environment in which their animals live.

In yet other situations, discrimination ability or salience may underlie apparent preference. For example, in a visual discrimination task zebrafish *Danio rerio* learned color cues very readily but not shape cues, until the shape cues were much enlarged (54). Here, a cue-use test led to the uncovering of a species' sensory abilities that had not been previously obvious. Yet more issues may be raised whereby the structure of the task itself has an impact on the animals' performance. For example, in an early examination of the neural bases of spatial learning in frogs, the animals were tested in a Morris water maze, a task in which the frogs, like rats, were thigmotaxic (keeping close to the edges of the pool). Frogs in that test did learn to use a visual cue to locate the platform hidden below the water's surface but would not swim across the center of the pool to reach it, even if that was the shortest distance to the platform (55). Rather, they swam around the edge and then used the pool wall to push off when they got close to the platform. If the authors had used the directedness of swim paths or speed to reach the platform exclusively to measure whether the frogs had learned the platform's location, as is typical in Morris water maze studies, these frogs would not have provided very convincing evidence that they could learn a spatial location. It is increasingly evident that frogs, like fish before them, are capable of learning spatial locations (56–59), a rather unsurprising confirmation if one considers the ecology of these species. Indeed, any animal that needs to find its way home, unless utterly dependent on volatile cues is likely to have some need for spatial memory. But then, examining cognition in frogs lags well behind even the work on fish.

## Sources of Cue Preferences

For anyone attending navigation conferences through the 1980's and 1990's (as was Braithwaite), the often-heated debate as to which was the primary cue used by pigeons to home between the Italian and German groups was a regular feature. The Italians argued that olfaction was key while the Germans argued that magnetic information was by far the more important. There was more than one accusation of poor science during such debates. What was needed was an experimental test. And when homing pigeons were experimentally raised in Frankfurt in the ‘Italian’ manner i.e., in a wind-exposed roof loft rather than in the typical-Frankfurt mode of an enclosed garden loft, these “Italian-Frankfurt” birds subsequently relied more heavily on olfactory than on magnetic information when homing (60), which was not the cue hierarchy of birds raised in the more standard Frankfurt manner. Importantly, this experiment provided rather good evidence that cue dependence seen in adults could depend very heavily on early experience, removing at least this point of contention from homing pigeon debates.

Given this background, when (61) investigated the sources of cue preference in sticklebacks, the obvious place to look was early environmental conditions in sticklebacks. Stickleback fry from pond and river populations, each raised with and without stable landmarks were tested in two ways: in a maze in which the fish needed to use visual landmarks to locate rewards and in an apparatus in which water flow was the relevant cue. The key result is that there was no difference in performance on either task between fish derived from river and pond populations. For all those working in the field of cognitive ecology, there were two important associated take-home messages from this result: (1) ecology can shape cue preference and use, but (2) a preference cannot be interpreted as evidence of an adaptation. Preferences are very likely to be at least somewhat flexible, and the nature of this flexibility may differ between species, or even between populations within a species.

One major source of flexibility, as shown by Girvan and Braithwaite, is the early environment, and particularly, the physical environment. Not only do fish pay attention to cues from their physical environment, but this early experience can also have a major effect on their capacity to cope with later-life complexities such as the release from hatchery conditions into the wild. Braithwaite, together with long-term collaborator Salvanes, showed that the provision of visual cues into tanks of juvenile cod can increase their reaction to novel prey and their speed to switch to natural, wild prey (62). They also showed that spatial structure in the early environment led to better anti-predator skills (63). These data on cod reared in hatcheries have not only led to a plethora of work conducted on cue use in an ecological context, but have also had a substantial and broad impact in both welfare and economic terms. A small sample of those contributions are more fully described and appreciated in other papers in this Special Issue.

More recent studies from Braithwaite's group have shown that the developmental stage of enrichment provision impactful [e.g., (64)], its duration [e.g., (65)], and its nature are all impactful. Braithwaite's focus was on the role that the physical, rather than the social, environment played on subsequent information use and learning [e.g., (66–68)]. She and colleagues also showed that at least zebrafish preferred an environment in which they could combine physical enrichment with swimming opportunities (66). Although many others had previously demonstrated impacts of physical and social enrichment on performance in learning and memory tasks of a wide range of species [e.g., (69, 70)], a recent meta-analysis (71) provides strong support for Braithwaite's own emphasis: asocial factors (physical enrichment, enclosure space, sensory enrichment, exercise) lead to larger impacts on learning than do social factors (isolation, parental deprivation, group size). Furthermore, duration of that enrichment also plays a major role, and apparently greater than the specific timing of the enrichment. Although the majority of the data on which this meta-analysis was based came from rodents [also now a meta-analysis on aquatic animals: Zhang et al. (72)], Braithwaite's work on fish is consistent with the broader taxonomic patterns.

## Non-Model Systems

No consideration of Braithwaite's work and its impact on the way we now regard fish cognition is complete without mention of her work using the tropical poecilid *Brachyraphis episcopi*. Like her stickleback work, working with Brachyraphis found Braithwaite out in the field collecting fish, but in this case, from streams along Pipeline Road, near Gamboa, Panama. The question to be addressed here was no longer the role that cues in the environment played in cognitive performance, but the role other species played, specifically that of predators. In the streams along Pipeline Road, Brachyraphis found above waterfalls typically share their stream with few fish species other than killifish *Rivulus brunneus*, while Brachyraphis living downstream below the waterfalls face a barrage of predators, the waterfalls being a considerable barrier to movement upstream for the downstream fish. Although boldness (speed of emergence from a shelter) does not always differ between fish from the two environments (73, 74), a variety of performance measures in a spatial task did: upstream (low predation) fish were more active within the maze, were faster to find the rewarded patch, and learned the rewarded location cue with fewer errors than did the fish from the high predation (downstream) sites (75). Recent tests of Trinidadian guppies *Poecilia reticulata* in the Lower and Upper Aripo (i.e., tested in the wild) showed that guppies from the high predation site were also less active and slower to complete a maze (76) and it would appear that at least some of these responses are learned in early life (77) and from parental behavior (78). Although begun with an interest in cognitive ecology, the Brachyraphis work then followed the growing enthusiasm for examination of individual differences and personality, leading to data on associations between environmental conditions and variation in aggression and boldness (79) and exploration (80). However, it never left cognitive ecology entirely behind, with the demonstration that Brachyraphis that explored more were also faster to learn to associate a cue with reward (81).

## Neural Work

No serious work on the role that natural selection plays on cognitive abilities (cognitive ecology) can avoid the part played by the brain. One example of this is the role of the hippocampus in the research on spatial in food storing mentioned above [e.g., in food-storing songbirds: Sherry and Vaccarino (43), Clayton and Krebs (82)]. In Braithwaite's own work, she and collaborators examined the role of neural plasticity in visual navigation in Atlantic salmon (67). This work showed that enrichment with physical landmarks that changed locations weekly led to enriched fish (Atlantic salmon) learning the correct exit from a simple maze with fewer mistakes than did the control fish. Examination of neural plasticity in the telencephalon (the part of the fish brain pertinent to spatial cognition) showed that enriched, but not control, salmon had upregulated expression of Neuro 1D mRNA expression. Just a few years on, it is becoming increasingly clear that environmental enrichment leads to neural cell proliferation (83–87) and it will now be interesting to determine which components of enrichment have this effect and why [e.g., (64)].

Evidence is also appearing for variation in brain regions involved in early stages of processing sensory information in recently diverged stickleback species. Limnetic species that are heavily reliant on visual information have larger optic tecta and smaller olfactory bulbs than do benthic species, which are much more dependent on olfaction (88). A similar effect is seen in killifish from sites with and without predation: killifish from sites with predators have large eyes and large optic tecta compared to killifish from sites without predators, but the whole brains of the two groups do not differ (89). Cognitive ecology in the round now has fish examples of the neurobiology of cognition to add to those from birds and mammals, a point reached in no small part thanks to Braithwaite and her collaborators.

## Not Just Fish

Finally, but very much not least, much as Braithwaite loved to work with and on fish, she was never just a “fish person”. She was always alert to systems that best addressed the question in which she was currently most interested. Braithwaite's continuing interest in spatial cognition led her first foray into examining spatial learning in rodents when she and collaborators examined the impact of parasitic infection (90). Her next and more substantial venture was inspired by the rich literature concerning the role of sex in spatial memory abilities in mammals, and especially rodents [e.g., (91–94)]. One feature of especial interest was the variation across this literature: some researchers found sex differences and some did not. When Braithwaite, together with a student and one of the current authors (Healy), collected some empirical data we also found no differences between the sexes in a spatial cognition task [Morris water maze (95)]. However, we did find that the number of swims the females needed to learn the location of the hidden platform performance differed across the 4 days of their oestrous cycles: they needed an extra swim on oestrous days. These data seemed to present a possible explanation for at least some of the cross-study variation as if females performance depends on the day on which they are tested, on some days they may perform as well, and on other days more poorly, than males.

Hormonal variation is a possible mechanistic explanation for sex differences in spatial cognition, but the question is why such hormonal differences would exist in the first place. There are also a multitude of evolutionary scenarios proposed to explain why the sexes might differ particularly in spatial cognition. Our subsequent consideration of the rationale and empirical data for the evolutionary explanations for differences between the sexes in spatial ability was, and still is, rather well-received by a greater diversity of fields than just evolutionary biology or animal cognition (96). Nearly 20 years later this review is being cited in such diverse work as bumblebee cognition (97), effects of binge drinking (98), stereotyped threat (99), gender differences in seminomadic pastoralist children (100), and behavior during COVID-19 lockdown in Russia (101). Although it has not led to policy changes as did her work on fish welfare (again see other papers in this Special Issue), the impact of this paper has been sustained and broad. Not bad for a paper that was addressed to a sideline interest.

## Legacy

When Les Real labeled cognitive ecology as an emerging field in 1993, he believed there was a sufficiently novel approach to deserve the name (35). Seven years later, present author Healy and Braithwaite wrote a cheekily early assessment asking if it was a field of substance (102). We wrote at the time that “there are those who will dispute the value of yet another label for yet another sub-discipline, and if little has happened in seven years, such critics will be right.” Now, over 20 years later, we can say with confidence that not only is cognitive ecology a field of substance, but that substance is in large part thanks to the work of Victoria Braithwaite. The field itself has truly begun to come of age with an increasing diversity of species under examination, in an increasing variety of contexts. Importantly, fish are now a mainstream taxon, along with mammals and birds, for addressing questions regarding cognition in general, and cognitive ecology specifically. This major change in the field is just one of Victoria's scientific legacies, and we know that she would look forward with keen interest to where the field will go next.

## Author Contributions

SH wrote much of the first draft. Both authors contributed to revisions.

## Conflict of Interest

The authors declare that the research was conducted in the absence of any commercial or financial relationships that could be construed as a potential conflict of interest.

## Publisher's Note

All claims expressed in this article are solely those of the authors and do not necessarily represent those of their affiliated organizations, or those of the publisher, the editors and the reviewers. Any product that may be evaluated in this article, or claim that may be made by its manufacturer, is not guaranteed or endorsed by the publisher.
